# Metallothionein 3 promotes osteoclast differentiation and survival by regulating the intracellular Zn^2+^ concentration and NRF2 pathway

**DOI:** 10.1038/s41420-023-01729-y

**Published:** 2023-12-01

**Authors:** Shinkichi Arisumi, Toshifumi Fujiwara, Keitaro Yasumoto, Tomoko Tsutsui, Hirokazu Saiwai, Kazu Kobayakawa, Seiji Okada, Haibo Zhao, Yasuharu Nakashima

**Affiliations:** 1 Department of Orthopaedic Surgery, Graduate School of Medical Sciences, Kyushu University, Fukuoka, Japan; 2 Department of Orthopaedic Surgery, Graduate School of Medical Sciences, Osaka University, Suita, Japan; 3 Southern California Institute for Research and Education, Long Beach, CA USA; 4 Center for Osteoporosis and Metabolic Bone Diseases, Division of Endocrinology, Department of Internal Medicine, University of Arkansas for Medical Sciences, Little Rock, USA; 5 Department of Physiology and Cell Biology, University of Arkansas for Medical Sciences, Little Rock, USA

**Keywords:** Differentiation, Targeted bone remodelling, Cell proliferation

## Abstract

In osteoclastogenesis, the metabolism of metal ions plays an essential role in controlling reactive oxygen species (ROS) production, mitochondrial biogenesis, and survival, and differentiation. However, the mechanism regulating metal ions during osteoclast differentiation remains unclear. The metal-binding protein metallothionein (MT) detoxifies heavy metals, maintains metal ion homeostasis, especially zinc, and manages cellular redox levels. We carried out tests using murine osteoclast precursors to examine the function of MT in osteoclastogenesis and evaluated their potential as targets for future osteoporosis treatments. MT genes were significantly upregulated upon differentiation from osteoclast precursors to mature osteoclasts in response to receptor activators of nuclear factor-κB (NF-κB) ligand (RANKL) stimulation, and MT3 expression was particularly pronounced in mature osteoclasts among MT genes. The knockdown of MT3 in osteoclast precursors demonstrated a remarkable inhibition of differentiation into mature osteoclasts. In preosteoclasts, MT3 knockdown suppressed the activity of mitogen-activated protein kinase (MAPK) and NF-κB signaling pathways upon RANKL stimulation, leading to affect cell survival through elevated cleaved Caspase 3 and poly (ADP-ribose) polymerase (PARP) levels. Additionally, ROS levels were decreased, and nuclear factor erythroid 2-related factor 2 (NRF2) (a suppressor of ROS) and the downstream antioxidant proteins, such as catalase (CAT) and heme oxygenase 1 (HO-1), were more highly expressed in the MT3 preosteoclast knockdowns. mitochondrial ROS, which is involved in mitochondrial biogenesis and the production of reactive oxygen species, were similarly decreased because cAMP response element-binding (CREB) and peroxisome proliferator-activated receptor γ coactivator 1β (PGC-1β) were less activated due to MT3 depletion. Thus, by modulating ROS through the NRF2 pathway, MT3 plays a crucial role in regulating osteoclast differentiation and survival, acting as a metabolic modulator of intracellular zinc ions.

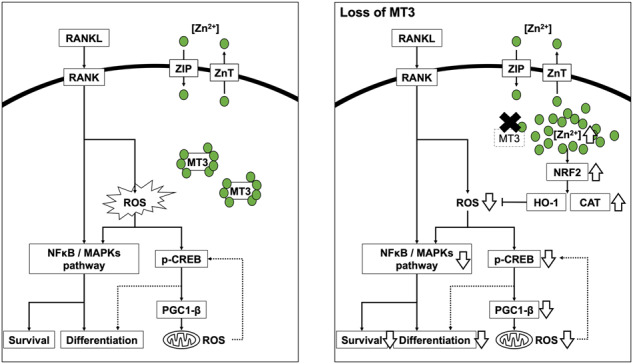

## Introduction

Osteoclast- and osteoblast-mediated bone formation is critical for skeletal development, homeostasis, and repair [1]. In adults, bone development and bone loss are intricately linked and balanced through a continuous self-renewal process known as bone remodeling that ensures that bone mass and strength are maintained throughout life [2, 3]. Increased osteoclast activity and numbers have been attributed to pathological bone loss and fragility fractures in metabolic bone illnesses such as osteoporosis, Paget’s disease, and osteolytic tumor metastases [4–6]. The cellular and molecular mechanisms governing osteoclast differentiation and operation in normal and pathological circumstances must be clarified. This understanding is essential for creating novel therapeutic approaches to cure bone loss in various skeletal illnesses efficiently.

Mononuclear progenitor cells are produced from the monocyte/macrophage lineage of hematopoietic stem cells, which fuse to form multinucleated cells called osteoclasts. The two cytokine receptor activators of nuclear factor-κB (NF-κB) ligand (RANKL) and macrophage colony-stimulating factor (M-CSF) are essential for osteoclastogenesis in vitro and in vivo [1, 7]. M-CSF primarily controls macrophage proliferation and osteoclast survival by stimulating the phosphoinositide-3-kinase/AKT (PI3K/AKT) and extracellular signal-regulated kinase (ERK) pathways [8, 9], while NF-κB, PI3K/AKT, and mitogen-activated protein kinase (MAPK) pathways are all activated by RANKL to control osteoclast differentiation [1, 4]. Furthermore, the main transcription factor for osteoclast differentiation, nuclear factor of activated T cells 1 (NFATc1), is induced by RANKL signaling [10], regulating calcium oscillation [7]. Metal ions such as calcium, iron, and zinc play pivotal roles in osteoclast differentiation and function. Primarily, iron homeostasis controls osteoclastogenesis and function via intracellular and mitochondrial reactive oxidative species (ROS) [11–14]. Moreover, zinc is essential for fostering the mineralization and development of bones by stimulating osteoblasts, and it exerts a suppressive effect on bone resorption by inhibiting osteoclast formation [15–17]. Thus, the metabolism of metal ions remains unknown mainly due to their complex roles.

The metal-binding protein, metallothionein (MT), which comprises high cysteine content (30%) and low molecular weight (6–7 kDa), has four primary isoforms (MT1-MT4) [18–20]. MT1 and MT2 are ubiquitously expressed in various soft tissues, while MT3 is primarily found in brain tissue, heart, kidney, and genital organs [21, 22]. MT4 is specifically expressed in certain squamous epithelia [23]. MT has a wide range of physiological functions, including heavy metal detoxification, maintenance of metal ion homeostasis (especially zinc and copper), regulation of cellular redox balance, and facilitation of cell proliferation [24–27]. In addition, the MT protein family has been implicated in pathological conditions such as cancer and neurodegenerative diseases [28, 29]. In mouse bone marrow stromal cells, MT serves a protective function against oxidative stress-induced suppression of osteoblast development. Additionally, a zinc-rich diet, which stimulates endogenous MT production, affects both osteoblast differentiation and postnatal bone growth [30, 31]. However, MT function in osteoclastogenesis is still unclear.

In this study, we identified MT3 as the dominant MT isoform expressed in osteoclasts, which was upregulated in murine and human osteoclasts after RANKL stimulation. A reduction of osteoclast differentiation was caused by the loss of MT3, which downregulated RANKL-stimulated MAPK/NF-κB signaling. Furthermore, loss of MT3 raised intracellular zinc concentrations, which in turn increased nuclear factor erythroid 2-related factor 2 (NRF2), an antioxidant defense mechanism, and decreased ROS in osteoclastogenesis. Our research suggests that MT3 is a new osteoclastogenesis regulator that controls ROS and the NRF2 pathway.

## Results

### Osteoclast development and bone resorption are inhibited in vitro by deleting MT3, which is abundantly expressed in murine and human osteoclasts

**Fig. 1:**
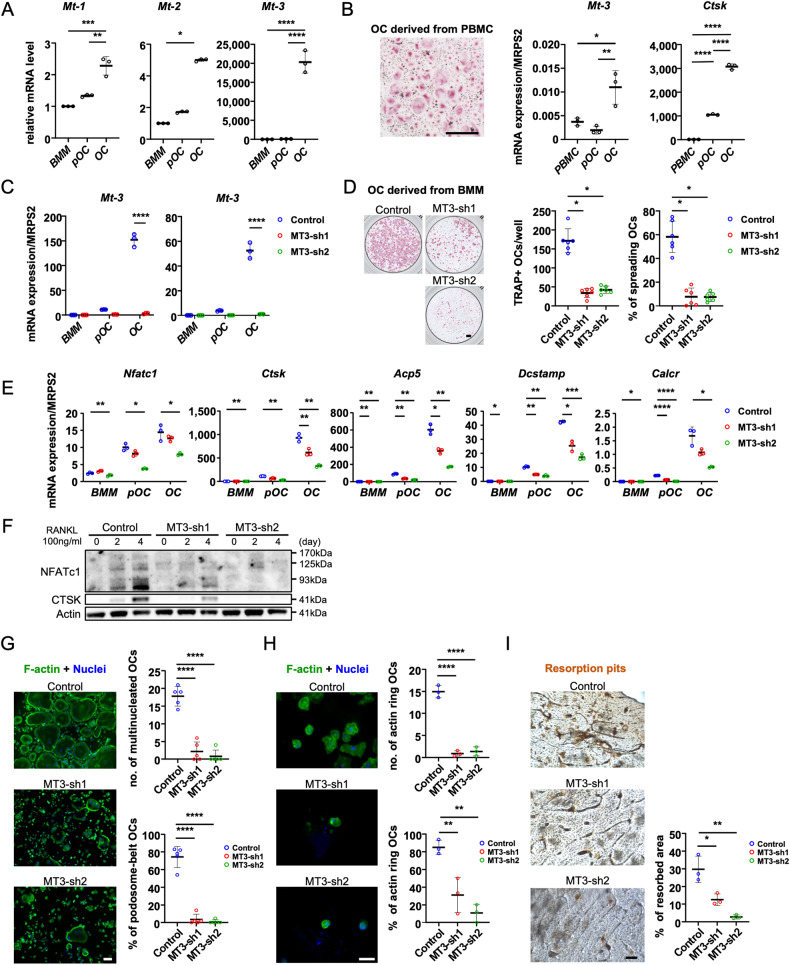
In osteoclast, MT3 is the most highly expressed in MT isoforms and is critical for osteoclast differentiation in vitro. **A** Bone marrow macrophages (BMM) were cultured with M-CSF, or with both M-CSF and RANKL for 2 and 4 days to generate preosteoclasts (pOC) and mature osteoclasts (OC), respectively. The mRNA expression levels of *Mt1-3* were measured using real-time PCR (*n* = 3). The graphs were presented by relative expression with BMM. **B** Human peripheral blood monocytes (PBMC) were cultured with M-CSF, or with both M-CSF and RANKL for 3 and 7 days to generate pOCs and OCs. TRAP staining of OC (scale bar = 500 μm) and the mRNA expression levels of human *Mt3* and osteoclast marker, cathepsin K (*Ctsk*), were examined (*n* = 3). **C** After knocking down BMM by MT3-shRNAs (MT-sh1 or -sh2) or a control shRNA (Control), the cells were induced to differentiate into osteoclasts for a duration of 4 days. The levels of *Mt3* expression were quantified using real-time PCR (*n* = 3). **D** TRAP staining and quantification of numbers of total and spreading OC cultured on plastic 96-well dishes (scale bar = 500 μm) (*n* = 6). **E** The mRNA expression levels of osteoclast markers, NFATc1 (*Nfatc1*); cathepsin K (*Ctsk*); TRAP (*Acp5*); DC-STAMP (*Dcstamp*); and calcitonin receptor (*Calcr*) were measured by real-time PCR (*n* = 3). **F** Protein expressions of osteoclast markers were detected by western blotting. Actin served as a loading control. **G** Actin filaments and nuclei were stained with Alexa-488-conjugated phalloidin and DAPI in osteoclasts cultured on glass coverslips (scale bar = 100 μm). The number of osteoclasts with different nuclei and the percentage of spreading osteoclasts with a peripheral podosome-belt were quantified (*n* = 5). **H** Actin filaments and nuclei were stained with Alexa-488 conjugated Phalloidin and DAPI in osteoclasts cultured on cortical bovine bone slices (scale bar = 50 μm). The number and the percentage of osteoclasts exhibiting an actin ring per bone slice were calculated (*n* = 3). **I** Resorption pits were stained by horseradish peroxidase-conjugated wheat-germ agglutinin (scale bar = 100 μm). The percentage of resorbed area per bone slice was calculated by Hybrid Cell Count Software (*n* = 3). The data are presented as mean ± SD. *p < 0.05; **p < 0.01; ***p < 0.001; ****p < 0.0001 vs control.

We first assessed the mRNA levels of *Mt1-3* with osteoclast formation using real-time quantitative PCR to determine the function of MT in osteoclast lineage cells. To achieve this, bone marrow macrophages (BMM) were cultivated with M-CSF alone or in combination with RANKL for 2 and 4 days for differentiation into preosteoclasts (pOC) and mature osteoclasts (mOC), respectively, each of which has a different function. Figure 1A demonstrates that during osteoclast development, the mRNA levels of *Mt1-3* increased, and *Mt3* had the highest expression among the *Mt* osteoclast isoforms compared with BMM, showing that Mt3 was the most prevalent isoform within the MT family during osteoclastogenesis. We also examined the expression of *Mt3* in human peripheral blood mononuclear cell-derived osteoclasts and found that its expression increased during osteoclastogenesis (Fig. 1B).

Our next goal was to define MT3’s cell-autonomous roles in osteoclast differentiation and function. To achieve this, we investigated if two short hairpin (sh) RNAs (MT3-sh1 and MT3-sh2) that targeted various murine Mt3 mRNA locations could knockdown MT3 expression in BMM via lentiviral transduction. The negative control was a shRNA directed against firefly luciferase. BMMs that had been positively transduced were either cultivated with M-CSF alone or for 3 days with M-CSF and RANKL. Both MT3-specific shRNAs, but not the control, significantly decreased *Mt3* mRNA expression in RANKL-induced mOC (Fig. 1C). Tartrate-resistant acid phosphatase (TRAP), a marker of osteoclast development, revealed a decrease in the number of multinucleated osteoclasts in the bone. Knockdown of MT3 in BMM inhibited osteoclast formation and significantly decreased the total osteoclast number and spreading area (Fig. 1D). Reduced mRNA expression of osteoclast marker genes, including NFATc1 (encoded by *Nfatc1*), cathepsin K (encoded by *Ctsk*), TRAP (encoded by *Acp5*), DC-STAMP (encoded by *Dcstamp*), and calcitonin receptor (encoded by *Calcr*), compared to control cells in MT3-depleted mOC (Fig. 1E) confirmed the reduction. Furthermore, MT3-depleted cells markedly decreased the protein expressions of NFATc1 and CTSK (Fig. 1F). We stained actin filaments using Alexa- 488 conjugated phalloidin in control and MT3 knockdown osteoclasts cultivated on plastic dishes and cortical bovine bone slices because actin cytoskeleton organization is crucial for osteoclast spreading and the production of podosome-belts on plastic plates (Fig. 1G) and actin-rings on bone matrix (Fig. 1H). Depleting MT3 in osteoclasts may impair their function because osteoclast activation and function are required for actin ring formation. In support of this hypothesis, MT3 knockdown osteoclasts exhibited less bone resorption than control osteoclasts, as shown by the staining of resorption pits on cortical bovine bone slices (Fig. 1I). These data suggest that MT3 is upregulated by RANKL and positively regulates osteoclast differentiation and function in vitro.

### MT3 is critical for osteoclast survival

**Fig. 2:**
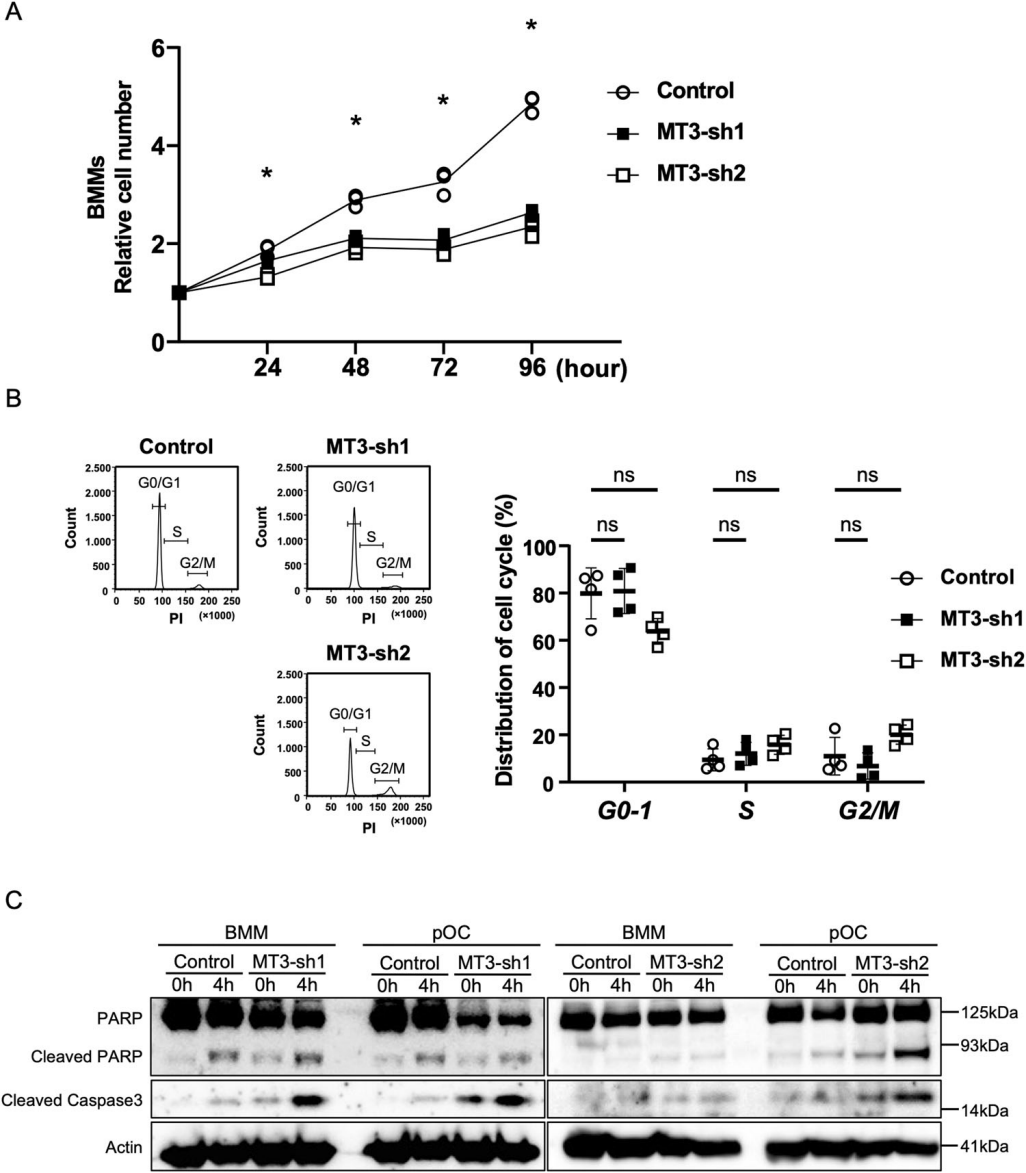
Loss of MT3 affected cell survival in preosteoclasts. **A** Relative cell number of Control and MT3-sh1/sh2 BMMs was presented up to 96 h in growth curve. The data are presented as mean (*n* = 3). *p < 0.001 vs control. **B** Representative histograms of cell cycle distribution in BMMs were measured by flow cytometry using Propidium iodide. The average percentage of cell numbers in each phase of the cell cycle is shown in the graphs. The data are presented as mean ± SD (*n* = 4). ns: no significance, vs control. **C** Control and MT3-sh1/sh2 of BMM or pOC were either untreated or serum/cytokine starved for 4 h. The levels of PARP and Cleaved Caspase 3, which are markers of apoptosis, were assessed using Western blot analysis. Actin served as a loading control.

To investigate the potential involvement of MT3 in BMM cell growth, we conducted a comprehensive analysis of cell growth over 96 h. MT3 knockdown by shRNA rescued the increased number of BMM compared with the control (Fig. 2A). Next, we attempted to determine which cellular processes were involved in the cell growth of osteoclast lineage cells after MT3 depletion. Figure 2B compares the cell cycle progression (S and G2/M) in BMM between control and MT3 knockdown groups using flow cytometry and reveals no discernible differences. The cell population in the G0-1 phase in control BMM or MT3 knockdown was also unchanged, suggesting that MT3 knockdown did not affect the BMM cell cycle progression. Western blotting of the apoptosis markers poly (ADP-ribose) polymerase (PARP) and cleaved (active) caspase 3 revealed that ablation of MT3 in BMM had a negligible impact triggered by cytokine or serum deprivation. Furthermore, knockdown of MT3 in pOC increased expression of cleaved PARP and cleaved caspase-3under the same circumstances (Fig. 2C). These results suggest that MT3 in osteoclast lineage cells accelerated osteoclastogenesis by affecting osteoclast survival.

### MT3 regulates osteoclastogenesis via activation of RANKL-induced JNK and NF-κB

**Fig. 3:**
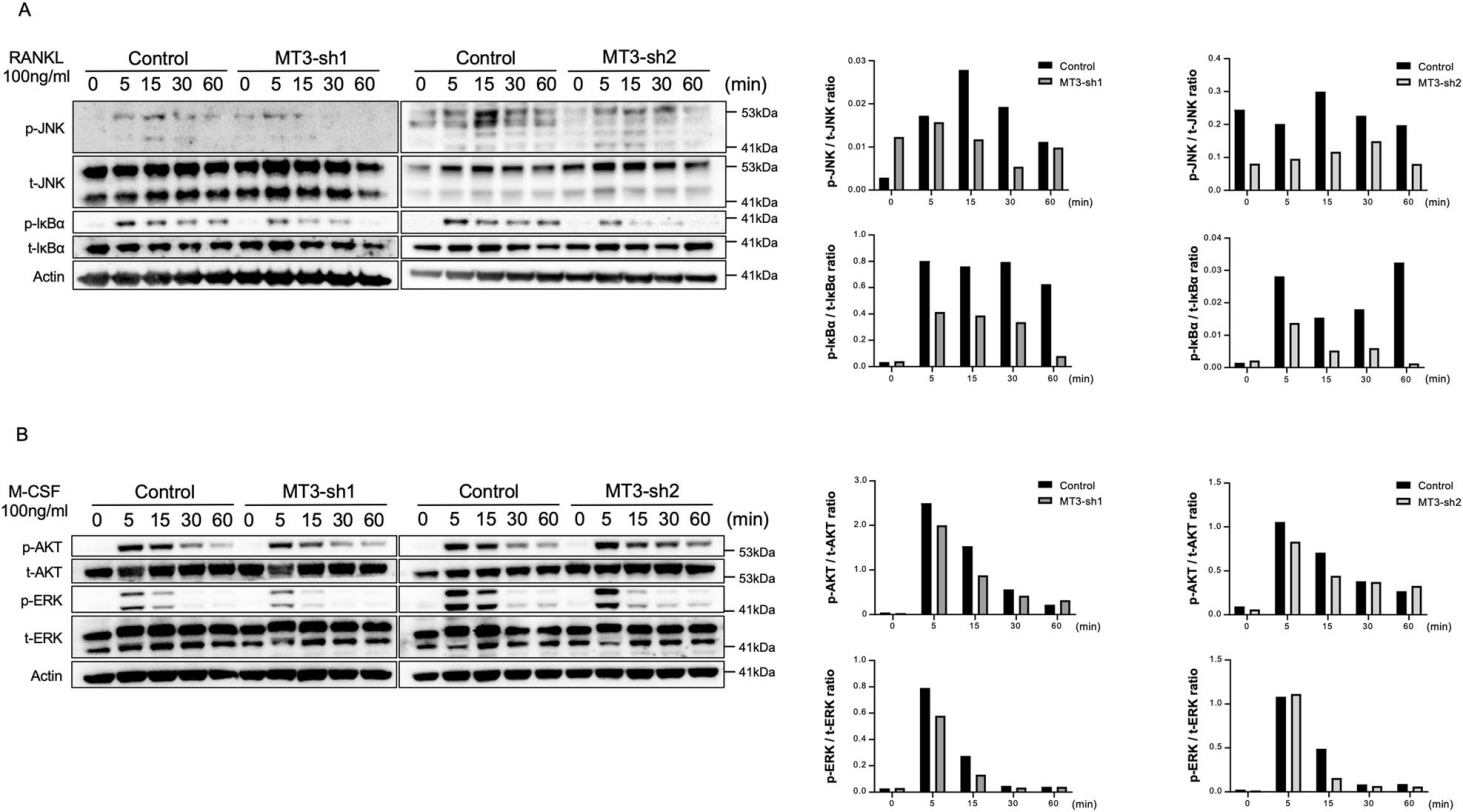
MT3 is required for RANKL-induced JNK and NF-κB activation in preosteoclasts. Western blot analysis (left) and quantification (right) detected the levels of phosphorylated and total JNK, IκBα, AKT, and ERK in pOC treated with RANKL (100 ng/mL) (**A**) or M-CSF (100 ng/mL) (**B**) for the indicated time. Actin served as loading controls.

M-CSF and RANKL signaling activation are essential for the proliferation, survival, and differentiation of osteoclastogenesis [7]. Control and MT3 knockdown preosteoclasts were starved with serum and cytokines and then stimulated with M-CSF or RANKL for the designated amount of time to determine which signaling in M-CSF or RANKL affected preosteoclasts of MT3 loss [32, 33]. Western blots were used to analyze the activation of c-Jun N-terminal kinase (JNK), NF-κB, and ERK, which are all induced by RANKL and M-CSF, respectively. AKT, also known as protein kinase B, and AK strain transformation are induced by M-CSF. Both MT3-shRNAs that knocked down MT3 in preosteoclasts had no impact on ERK and AKT phosphorylation in response to M-CSF stimulation (Fig. 3B). In contrast, JNK and NF-κB activation caused by RANKL was consistently reduced by MT3 deletion by both shRNAs, evidenced by lower levels of phospho-JNK and phospho-IκB (Fig. 3A). Given that MT3 regulates the JNK and NF-κB pathways, which are essential for osteoclast differentiation and survival, these findings imply that MT3 modulates RANKL-induced activation of JNK and NF-κB, which controls osteoclast differentiation and survival [5].

### The loss of MT3 triggers the activation of the NRF2 pathway, leading to the inhibition of RANKL-induced ROS production

**Fig. 4:**
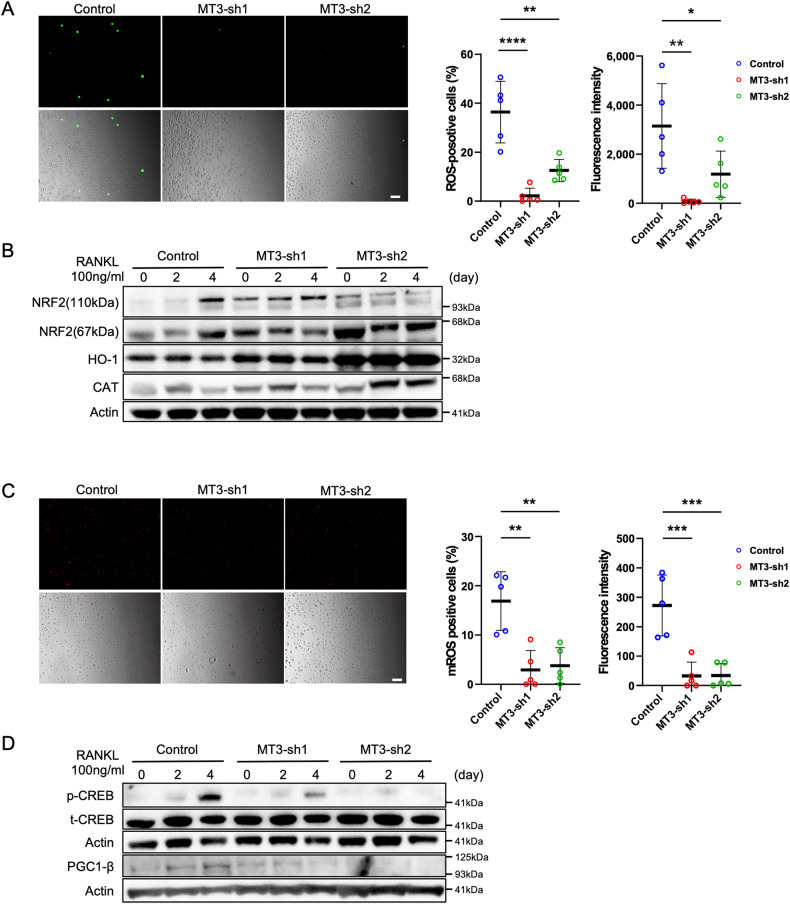
Change of RANKL-induced ROS and mitochondrial ROS levels by depletion of MT3 expression. **A** Representative images of intracellular ROS levels in pOCs were measured by DCFH-DA assay followed by RANKL stimulation for 48 h (scale bar = 100 μm) and the percentage of positive cells and the fluorescence intensity on cells of each well were calculated by Hybrid Cell Count Software (Keyence) (*n* = 5). **B** The expressions of NRF2, HO-1, and CAT were detected by western blots. Actin served as a loading control. **C** Representative images of mitochondrial ROS levels in pOCs were measured by MitoSOX Red Mitochondrial Superoxide Indicator, followed by RANKL stimulation for 48 h (scale bar = 100 μm) and the percentage of positive cells and the fluorescence intensity on cells of each well were calculated, respectively (*n* = 5). **D** The expressions of pCREB and PGC-1β were detected by western blots. t-CREB and Actin served as loading controls, respectively. The data are presented as mean ± SD. *p < 0.05; **p < 0.01; ***p < 0.001; ****p < 0.0001 vs control.

In osteoclastogenesis, RANKL-induced activation of MAPKs (ERK, JNK, and p38), NF-κ-B, and the PI3K/AKT pathways depends on ROS generated by RANKL and MT has a protective role against oxidative stress [34–37]. So, we examined the production of ROS during osteoclastogenesis by DCFH-DA staining. As shown in Fig. 4A, deletion of MT3 in preosteoclasts dramatically decreased intracellular ROS production by assessing the positivity rate of DCF fluorescence, indicating that depletion of MT3 reduced ROS production induced by RANKL during osteoclastogenesis. ROS act as mediators of intracellular signaling involved in the development and activation of osteoclasts in response to RANKL stimulation [34, 38, 39]. Additionally, nuclear factor erythroid 2-related factor 2 (NRF2) is a defensive mechanism against oxidative stress that regulates several antioxidant enzymes, such as NAD(P)H quinone dehydrogenase 1 (NQO1), catalase (CAT), haemoxygenase-1 (HO-1), and γ-glutamyl cysteine synthetase (GCS) [40–43]. The overexpression of NRF2 suppresses osteoclastogenesis in vitro and in vivo due to the reduction of oxidative stress. To investigate the association with ROS-related enzymes in MT3 loss, the expression of ROS-related enzymes, including CAT, HO-1, and NRF2, with osteoclast differentiation is presented in Fig. 4B by western blotting. In comparison to the control, MT3 knockdown considerably boosted the expression levels of NRf2 and its downstream antioxidant factors, HO-1 and CAT, in BMM and pOC, leading to less ROS production during osteoclast differentiation. These findings may provide mechanistic evidence that RANKL-induced intracellular ROS production is effectively attenuated by the loss of MT3, which upregulates NRF2, and the associated downstream antioxidant enzymes.

### Depletion of MT3 decreased mitochondrial ROS levels in osteoclast precursors

ROS activates the downstream mediator peroxisome proliferator-activated receptor coactivator 1β (PGC-1β) via phosphorylating the cAMP response element-binding protein (CREB) during osteoclastogenesis, stimulating mitochondrial biogenesis, and the generating of mitochondrial ROS, which in a positively feedback-induced manner increases CREB and PGC-1β activity and facilitates NFATc1 activation [11, 12, 44, 45]. The deletion of MT3 in preosteoclasts also diminished mitochondrial ROS levels, similarly to intracellular ROS production (Fig. 4C). We then analyzed CREB phosphorylation and PGC-1β expression in cells treated with MT3 shRNA to promote osteoclastogenesis, which resulted in a reduction of CREB phosphorylation and PGC-1β expression in the depletion of MT3 osteoclasts (Fig. 4D). These data suggest that MT3 regulates mitochondrial ROS levels via the activation of CREB and PGC-1β.

### MT3 loss led to the accumulation of intracellular Zn^2+^, potentially leading to the upregulation of NRF2 gene expression

**Fig. 5:**
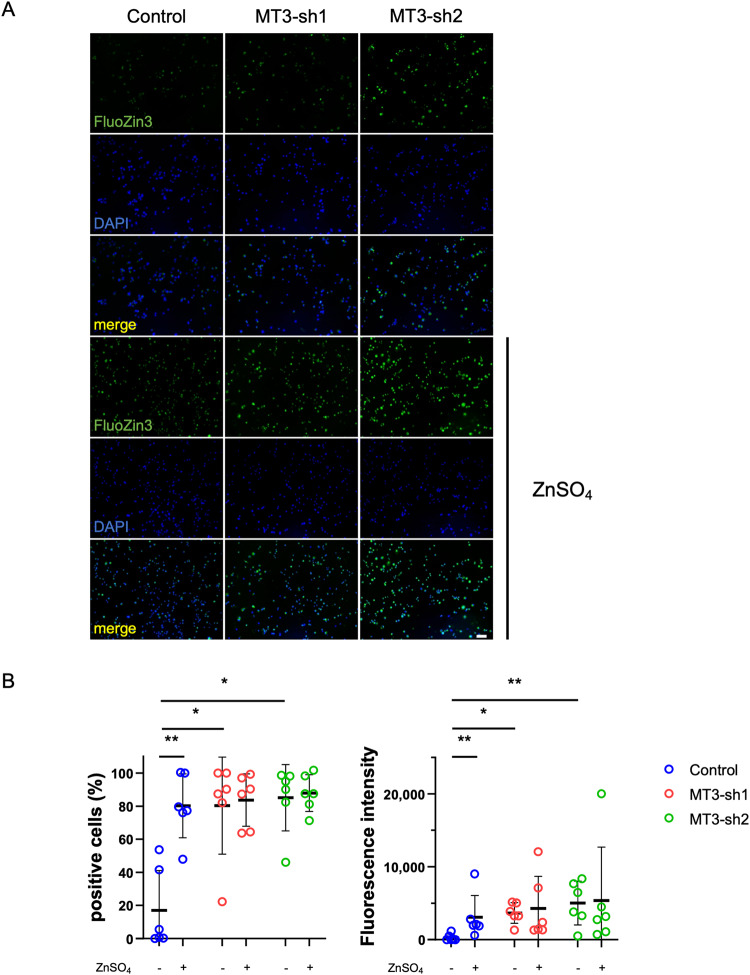
The levels of intracellular Zn^2+^ were increased in the MT3 knockdown pOC. **A** Confocal fluorescence microscopic images of pOC of Control and MT3-sh1/sh2 stained with FluoZin-3 (green) and DAPI (blue). Samples were treated and untreated with 50 μM ZnSO_4_ for 1 h (scale bar = 100 μm). **B** The percentage of positive cells and the fluorescence intensity on cells were calculated by Hybrid Cell Count Software (*n* = 6). The data are presented as mean ± SD. *p < 0.05; **p < 0.01 vs control.

**Fig. 6:**
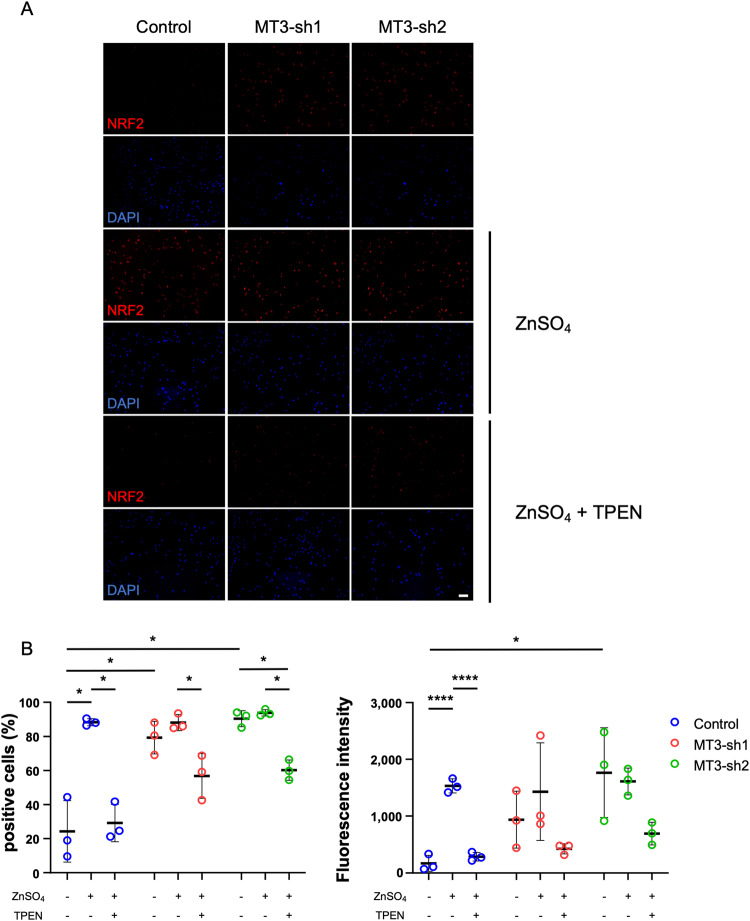
The expression of NRF2 was upregulated by MT3 knockdown, increased by zinc administration, and suppressed by zinc chelation. **A** Immunofluorescence assay of NRF2 (red) and DAPI-labeled nuclei (blue) treated and untreated with 50 μM ZnSO_4_ and 5 μM TPEN in pOc (scale bar = 100 μm). **B** The percentage of positive cells and the fluorescence intensity were calculated by Hybrid Cell Count Software (*n* = 3). The data are presented as mean ± SD. *p < 0.05 vs control.

MT acts as a biochemical regulator of intracellular levels of free Zn^2+^ by capturing and subsequently releasing Zn^2+^ in response to various biochemical events, such as oxidative signaling [46]. Visualization using FluoZin-3 fluorescence microscopy verified that preosteoclasts without MT3 resulted in an elevation of intracellular Zn^2+^ levels at baseline conditions. Furthermore, intracellular levels of Zn^2+^ were enhanced 1 h after the administration of 50 μM ZnSO_4_ in the MTs-depleted cells (Fig. 5). The change in expression of NRF2 in preosteoclasts after treatment with Zn^2+^ was examined with or without MT3 because it has been reported that Zn^2+^ is essential for NRF2 expression and transcriptional function [47–51]. As shown in Fig. 6, MT3 depletion in preosteoclasts showed higher intracellular NRF2 expression, consistent with western blotting (Fig. 4B). After administration of ZnSO_4_, NRF2 expression was increased in MT3 knockdown cells than controls. However, this effect was treated with N, N, N′, N′-tetrakis-(2-pyridylmethyl) ethylenediamine (TPEN), a cell-permeable zinc chelator, and was successfully removed (Fig. 6), suggesting that MT3 loss elevated NRF2 expression by the accumulation of intracellular Zn^2+^.

## Discussion

**Fig. 7:**
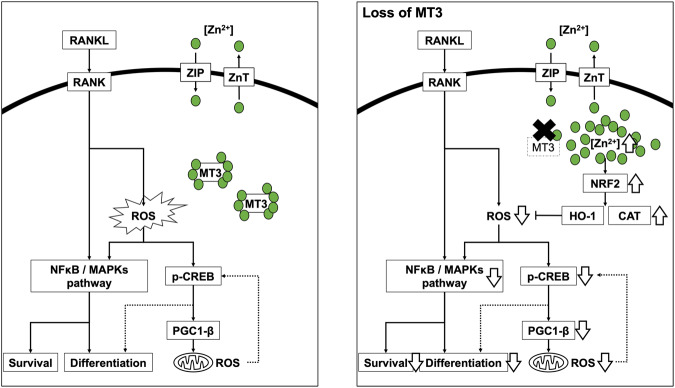
Schematics of signaling pathways and a model of MT3 function in osteoclastogenesis.

Increased intracellular ROS production in osteoclastogenesis encourages cell survival and differentiation through the activation of MAPK and NF-κB. It is also implicated in the generation of mitochondrial ROS, which is mediated by pCREB and PGC-1β [12, 44]. In particular, the metabolism of cellular metal ions, such as iron and copper, regulates ROS production and mitochondrial biogenesis [12, 14, 52, 53]. However, the molecular processes governing cellular metal ions in osteoclastogenesis are still unclear. This study elucidated that the metal-binding protein MTs, particularly MT3, were upregulated during osteoclast differentiation of murine and human osteoclasts. MTs have various physiological functions, such as detoxification of heavy metals, maintenance of metal ion homeostasis (particularly Zn^2+^), and regulation of cellular redox balance [24–27]; However, MT3 suppression prevented osteoclast development and affected cell survival in osteoclast precursor cells. This effect was linked to increased free intracellular Zn^2+^ that was not bound to MT3 and led to the downregulation of ROS production by increasing the expression of NRF2, HO-1, and CAT. The decreased ROS resulted in reduced activation of MAPK, NF-κB, pCREB, and PGC-1β, resulting in decreased survival and differentiation of osteoclasts (Fig. 7).

MT controls the redox reactions and forms the redox complex with Zn^2+^, which scavenges and neutralizes free radicals via cysteine sulfur ligands and donates Zn^2+^ in a redox-dependent manner [35–37]. Although MT3 is specifically expressed in the central nervous system, it has been recently observed to have various functions in other tissues [19]. MT3 regulates ROS by controlling metal metabolism such as zinc and cooper. The suppression of ROS could be effective in inhibiting RANKL-induced osteoclastogenesis given that RANKL activates MAPK and NF-κB, causing ROS to enhance osteoclastogenesis and associate with the pathogenesis of osteoporosis [34, 45]. In this study, MT3 knockdown in osteoclast progenitor cells decreased JNK activation, and ROS production, and RANKL-induced NF-κB stimulation, suggesting that MT3 may affect osteoclast survival and differentiation by regulating ROS and downstream MAPK and NF-κB signals. Therefore, to investigate the association with MT3, ROS, and osteoclastogenesis, we focused on the metabolism of metals, especially zinc.

The presence of zinc is essential for the antioxidant action of MT. After exposure to oxidative stress, glutathione (GSH) neutralizes free radicals by donating hydrogen to form glutathione disulfide (GSSG). Glutathione reductase can reconvert GSSG to GSH to continue the redox cycle in the presence of intracellular free Zn^2+^-bound MT [54]. Zinc is known to promote bone formation and suppress bone resorption [15–17] via inhibition of osteoclast differentiation induced by phosphorylation of calcineurin [55], inhibition of NF-κB signaling [56], and enhancement of apoptosis [57]. MTs bind and release Zn^2+^ under physiological conditions and are part of a network that tightly regulates intracellular Zn^2+^ in concert with the cell-specific Zn^2+^ importer, Zrt- and Irt-like proteins (ZIP) and Zn^2+^ exporter, Zn transporters (ZnT). When the free intracellular Zn^2+^ concentration reaches a threshold, the activation of metal-responsive transcription factor 1 induces MT expression, which sequesters Zn^2+^ and releases it in response to other biochemical events, such as oxidative signals, leading to the maintenance of homeostasis of free Zn^2+^ [46, 58]. In this study, it is possible that the knockdown of MT3 failed to sequester intracellular Zn^2+^ and increased intracellular zinc concentration. In support of this result, Malaiyandi et al. [59] and Habel et al. [60] demonstrated that MT overexpression reduced the concentration of intracellular Zn^2+^. This study has not shown overexpression control of MT3 in BMM. Previous report has indicated that the overexpression of MT3 results in zinc depletion, attributed to the dysregulation of zinc finger transcription factors and other zinc-requiring proteins, with consequent effects on cell growth, differentiation, and programmed cell death [61].

NRF2, which is induced by Zn^2+^, plays a central role as a part of the antioxidant defense system of osteoclasts in reducing ROS [62, 63]. NRF2, as a redox-sensitive transcription factor, expresses antioxidant enzymes against oxidative stress and inflammatory responses and suppresses RANKL-induced osteoclastogenesis [42, 43]. NRF2 suppresses ROS and NFATc1 and PGC-1β directly [64]; the NRF2 activator is a powerful therapeutic target [5, 65, 66]. In our study, MT3 knockdown activated NRF2 at the stage of BMM and preosteoclasts compared with control cells. HO-1 and CAT, which are downstream factors, were also activated, indicating that NRF2 signals suppressed ROS production. Indeed, NRF2/Kelch-like ECH-associated protein 1 (KEAP1) pathway, which scavenges ROS, promotes NF-κB and NFTAc1 and regulates osteoclastogenesis in vitro and in vivo, suggesting that NRF2 activators as a new therapeutic target of osteoporosis [67–69]. Furthermore, as described above, zinc has been reported to be involved in activating NRF2 [47–51]. We also demonstrated that NRF2 was upregulated in MT3 knockdown, with elevated Zn^2+^ in preosteoclasts, and inhibition of intracellular Zn^2+^ by TPEN decreased NRF2 expression. These results suggest that the increased concentration of intracellular Zn^2+^ may be involved in the upregulation of NRF2 in preosteoclasts.

During osteoclastogenesis, RANKL-induced ROS induced PGC-1β via activation of CREB, and PGC-1β increased the production of reactive oxygen species and mitochondrial biogenesis, leading to the induction of osteoclastogenesis through a positive feedback mechanism [11, 12, 44, 45]. In addition, NRF2 activity suppresses ROS production and directly reduces PGC-1β, resulting in a lower expression level of mitochondrial genes [11]. Subsequently, the expression of IRF8, an NFATc1 antagonist, is upregulated and inhibits NFATc1 activation and osteoclast differentiation [64]. Our results also showed that the deletion of MT3 reduced the activation of CREB, PGC-1β, and mitochondrial ROS, suggesting that the reduction of ROS associated with NRF2 activity due to MT3 knockdown may lead to the inhibition of osteoclast differentiation through this mechanism. These data suggest that MT3 regulates osteoclastogenesis through NF-κB and/or CREB in osteoclast precursors. To support this hypothesis, direct evidence could be obtained by overexpressing NF-κB or CREB in MT3 knockdown cells and demonstrating the rescue of osteoclastogenesis defects. Nonetheless, conducting this experiment faces a current technical challenge as primary BMMs have shown difficulty surviving two rounds of viral transduction (unpublished data). Future experiments using MT3-null macrophages isolated from MT3 knockout mice will be very helpful.

In osteoblast, zinc supplementation, which induces intracellular MT expression, has demonstrated the capacity to enhance osteoblast differentiation [31, 70]. During BMP4-induced differentiation of C2C12 myoblast cells into osteoblasts, MT3 showed high expression level among different MT isotypes and actively facilitated differentiation by reducing ROS levels [71]. However, Mandal et. al has reported that ROS generation promotes BMP2-induced osteoblast differentiation in 2T3 pre-osteoblasts [72], and Mody et al. has presented that oxidative stress enhances osteoblast differentiation in vascular smooth muscle cells [73], indicating that the role of ROS in osteoblast differentiation dependents of cell type or cellular environment. In addition, MT3 germline knockout (KO) mice have been evaluated in the field of the neurological disorders, and have impaired social interactions but normal spontaneous motor activity, habituation, learning and memory [74]. Moreover, in both MT1-2 germline KO mice, it has been reported that zinc and MT1-2 are required for bone growth and MT3 is upregulated in the absence of MT1-2 [31]. In order to better understand the function of MT3 in osteoclasts and osteoblasts, it is important to evaluate cell-specific MT3 conditional knockout mice in the future.

We suggest that RANKL elevates MT3 with osteoclast development based on the findings of this investigation and those published in the literature. MT3 regulates intracellular Zn^2+^ activity, leading to ROS production regulation via the NRF2, HO-1, and CAT pathways. Subsequently, ROS activation possibly promote RANKL-induced MAPK and NF-κB, enhancing osteoclastogenesis. In addition, MT3 regulates mitochondrial ROS by activating PGC-1β and CREB (Fig. 7).

## Materials and methods

### Reagents and antibodies

The following antibodies were used: Nfatcl (#sc-7294) and catalase (#sc-271803), all of which were bought from Santa Cruz Biotechnology (Dallas, TX, USA); Actin (#A00702), from GeneScript (Piscataway, NJ, USA); cathepsin K (#MAB3324), purchased from EMD Millipore Corporation (Temucula, CA, USA); PARP (#9542), cleaved Caspase 3 (#9664), JNK/SAPK (#9252), phosphor-JNK/SAPK (#9251), IκBα (#9242), phosphor -IκBα (#9246), AKT (#2920), phosphor-AKT (#4058), phosphor-ERK1/2 (#9106), ERK1/2(#9102), CREB (#4820), phosphor-CREB (#9191) were bought from Cell Signaling Technology (Beverly, MA, USA); NRF2 (#A0674) were bought from ABclonal (Wuhan, Hunan, China); HO-1 (#ab189491), PGC-1β (#ab176328) were purchased from Abcam (Cambridge, MA, USA). α-Minimum Essential Medium (α-MEM) and Penicillin-Streptomycin Solution (×100) were bought from FUJIFILM Wako (Osaka, Japan), and fetal bovine serum (FBS) was purchased from Gibco (Billings, MT, USA).

### Animal use approval

The Institutional Animal Care and Use Committee at Kyushu University approved the study’s research techniques, which used mice. Furthermore, the National Institutes of Health’s recommendations for the moral treatment and use of animals were strictly followed in all the research that was carried out.

### BMM and osteoclast cultures

BMMs were prepared following the previously described method [32]. The tibia and femurs of 8–10-week-old C57/BL6J mice were extracted for the entire bone marrow. A lysis buffer (150 mM NH4Cl, 10 mM KNCO_3_, 0.1 mM EDTA, pH 7.4) was used for 5 min at room temperature to remove red blood cells. Then, for the next 4–5 days, 5 × 10^6^ bone marrow cells were plated onto a 100 mm petri dish and cultured in α-10 medium (α-MEM, 10% heat-inactivated FBS, 1 penicillin–streptomycin) with an addition of 1/10 volume of CMG 14-12 (conditioned medium supernatant containing recombinant M-CSF at 1 μg/ml) [75]. Every other day, the CMG 14-12 supernatant, and culture media were replaced. After 3 and 5 days of BMM culture (at a density of 160/mm), preosteoclasts and osteoclasts were produced by adding 1/100 volume of CMG 14-12 culture supernatant and 100 ng/ml of recombinant RANKL (Oriental yeast, Tokyo, Japan), respectively.

### Human osteoclast cultures

Using Ficoll-PaqueTM PLUS (Cytiva, Uppsala, Sweden), human peripheral blood monocytes (PBMCs) were separated from the blood of a healthy volunteer. Monocytes were purified from PBMCs using CD14 MicroBeads, human (Miltenyi Biotec, North Rhine-Westphalia, Germany) as per the guidelines provided by the manufacturer. To differentiate human osteoclasts, monocytes of human PBMC origin were cultured for 7 days (at a density of 3000/mm) in α-10 media containing 50 ng/ml of recombinant human M-CSF (Abcam, #ab259396) and 100 ng/ml of recombinant RANKL. Every 3 days, new media, and cytokines were replenished. This study adheres to the principles outlined in the Declaration of Helsinki. The study protocol received approval from the Regional Committee of Ethics for Human Research at the Faculty of Medicine, Kyushu University (22314-00). Prior to their involvement in the study, all participants provided their informed consent by signing appropriate documentation.

### Lentivirus mediated shRNA expression

ShRNA expression is mediated by lentiviruses. ShRNA targeting the mRNA of murine MT3 is expressed by the pLKO.1 lentiviral vector [TRCN0000257921/NM_013603.1-200s21c1 (MT3-sh1: 5′-CTGTGTGTGCAAAGGTGAAGA-3′) and TRCN0000249518/NM_013603.1-93s21c1 (MT3-sh2: 5′-TCCTGCACCTGCTCGGACAAA-3′) were purchased from Sigma-Aldrich]. The control was pLKO.1 puro nontarget shRNA control transduction particles (07181827MN). Using the TransIT transfection reagent (Mirus), 293-T cells were co-transfected with an pLKO.1 gene transfer vector and the virus packaging vectors ΔH8.2 and VSVG. After 48 h of transfection, virus supernatants were collected. The virus supernatant was used to transduce bone marrow-derived macrophages (BMMs), including M-CSF and 20 g/ml of protamine (Sigma-Aldrich). The transduced cells were subsequently chosen for 3 days in an α -10 medium supplemented with M-CSF and 60 µg/ml of puromycin (Sigma-Aldrich) [76, 77].

### TRAP staining

On a 48-well tissue culture plate, TRAP stains, BMMs were grown for 4–5 days in an α-10 medium containing M-CSF and RANKL. Following the culture, the cells were fixed using a solution of 4% paraformaldehyde (Wako) and phosphate-buffered saline (PBS). According to the previously reported procedure, TRAP staining was carried out using NaK tartrate and Naphthol AS-BI phosphoric acid (Sigma-Aldrich) [32].

### Quantitative real-time RT-PCR and RNA isolation

Quantitative real-time RT-PCR and RNA isolation were performed following the instructions provided by the manufacturer. Total RNA was purified using the RNeasy mini kit (Qiagen, Hilden, Germany). According to the manufacturer’s instructions, the Prime-ScriptTM RT reagent kit (Takara Bio, Kusatsu, Japan) was used with 0.5–1 μg of total RNA for synthesizing first-strand cDNAs. Using the following: primers from Thermo-Fisher Scientific, TaqMan quantitative real-time PCR was carried out: Mt-1 (Mm00496660_g1), Mt-2 (Mm00809556_s1), Mt-3 (Mm00496661_g1, Hs00359394_g1), Ctsk (Mm00484039_m1, Nfatc1 (Mm00479445_m1), Hs00166156_m1), Acp5 (Mm00475698_m1), Dcstamp (Mm01168058_m1), Calcr (Mm00432282_m1), Mrps2 (Mm03991065_g1 Hs00211443_m1). Thermo-Fisher Scientific ABI QuantStudio3 equipment was utilized for the amplification of the samples. Denaturation at 95 °C for 10 min was the first stage in the amplification process. This was followed by 40 cycles of denaturation at 95 °C for 15 s and annealing/extension at 60 °C for 1 min. Normalization was performed using the ΔCt method [78] to determine the relative cDNA amount, with the expression level of mitochondrial gene Mrps2 serving as the reference, whereby both BMMs, and osteoclasts show steady expression. The delta ΔCt technique was used to analyze the relative amounts of MT3 cDNAs in BMMs. Every test was run in triplicate.

### Immunoblotting

After two ice-cold PBS washes, cultured cells were lysed using Cell Lytic M (Sigma-Aldrich), which contained Phosphatase Inhibitor Cocktail (ab201112, Abcam) and Protease Inhibitor (cOmplete Mini, EDTA-free, Sigma-Aldrich). The cell lysates were centrifuged at 14,000 rpm for 15 min at 4 °C to remove cellular debris after being incubated on ice for 30 min. Polyacrylamide gels with a 4–12% gradient were loaded with a total of 10–30 micrograms of total protein (Invitrogen, Carlsbad, CA, USA) and electrophoretically transferred onto a polyvinylidene difluoride membrane (Amersham Biosciences, Arlington Heights, IL, USA) using a semi-dry blotting system (Bio-Rad, Hercules, CA, USA). The membrane was next blocked in 5% fat-free milk/Tris-buffered saline for an h before being treated with primary antibodies overnight at 4 °C (Santa Cruz Biotechnology). Finally, secondary antibodies conjugated with horseradish peroxidase were applied to the membrane. Immunoreactivity was detected using ECL Prime (Amersham Biosciences) and photographed using an Ez Capture MG (ATTO, Tokyo, Japan) after three washings with Tris-buffered saline containing 0.1% Tween 20. Band densities were calculated using CS Analyzer 3.0 (ATTO).

### Fluorescent staining of actin filament and nuclei

Actin filament and nuclear fluorescent staining osteoclasts cultivated on glass coverslips were permeabilized with 0.2% Triton X-100/PBS for 10 min at room temperature and fixed with 4% paraformaldehyde (Wako) in PBS for 20 min. For 15 min at room temperature, Alexa-488 conjugated phalloidin (1:100 from a 1 mg/ml stock) was used to label filament actin. Slow Fade Diamond Antifade Mountant with DAPI (Invitrogen) was used to stain nuclei following two 5-min PBS washes.

A fluorescence microscope (BZ-X810; Keyence, Osaka, Japan) was used to take pictures of the samples. The mean number of active osteoclasts (podosome-belt bearing osteoclasts on glass coverslips and actin-ring bearing osteoclasts on bone slices) with different nuclei and the percentage of spreading osteoclasts were calculated by Hybrid Cell Count Software using images of five randomly selected areas on each glass coverslip or bone slice.

### Resorption pit staining

Fourr percent paraformaldehyde (Wako)/PBS was used to fix mature osteoclasts cultured on cortical bovine bone slices for 20 min cells were eliminated from bone slices using a soft brush after PBS was washed twice for 5 min. The slices were then treated for 60 min at room temperature with 20 μg/ml peroxidase-conjugated wheat-germ agglutinin lectin. After washing twice in PBS, bone chips were treated with 0.03% H_2_O_2_ and 0.52 mg/ml 3,3-diaminobenzidine for 30 min. Samples were imaged using a fluorescent microscope (BZ-X810; Keyence, Osaka, Japan) and mounted in 80% glycerol/PBS [79]. Hybrid Cell Count Software was used to determine the mean percentage of resorbed area in each bone slice after collecting blinded photographs of five randomly selected locations per bone slice.

### Viability assay

In 96-well plates, cells were seeded at 5.0 × 10^3^ per well. The vitality of cells in each well was assessed as a reference value after four h of seeding. The CellTiter-Glo Luminescent Cell vitality kit (Promega, Madison, WI, USA) was then used to check the relative cell vitality every 24 h for a total of 96 h.

### Flow cytometric study of the cell cycle

Trypsin was used to collect the cells, which were then washed with PBS before being fixed in ice-cold 70% ethanol at 4 °C for 30 min. The cells were fixed, washed with PBS, and then treated with propidium iodide and RNase (Immunostep S.L., Salamanca, Spain) for 15 min. Flow cytometry was conducted using BD FACS Verse (Becton, Dickinson and Company, Franklin Lakes, NJ, USA), and the data were analyzed using BD FlowJo Software.

### ROS detection

2,7-Dichlorodihydrofluorescein diacetate (DCFH-DA) was used to measure the production of ROS (Cayman, Michigan, USA) as a fluorescent probe. Briefly, in an FBS-free medium with DCFH-DA (final concentration of 50 µM), cells from various groups were grown and incubated for 30 min. Subsequently, to remove extra DCFH-DA, cells were washed three times in serum-free media. A BZ-X800 microscope was used to take the fluorescence images, and the percentage of cells exhibiting positive fluorescence and the fluorescence intensity were quantified and analyzed using Hybrid Cell Count Software.

### Mitochondrial ROS production measurements

We worked with (Thermo-Fisher Scientific’s) MitoSOX Red Mitochondrial Superoxide Indicator to examine the generation of mitochondria-derived ROS. In brief, MitoSOX was incubated with cells in glass-bottom imaging dishes for 15 min at 37 °C at a concentration of 5 μM. Following this, Hanks’ buffer was used to wash the cells three times to eliminate any remaining probe, and then the cells were examined using a fluorescent microscope. Hybrid Cell Count Software examined the percentage of positive cells and the fluorescence intensity.

### The levels of intracellular Zn^2+^ measurements

In 24-well plates, 3 × 10^4^ cells were planted, and the cells were then treated with M-CSF and RANKL for 2 days. Subsequently, the cells were stained with FluoZin-3 (Invitrogen) at a concentration of 10 μM for 30 min. Following staining, Hanks’ buffer was used to wash the cells twice and fix them with a 4% paraformaldehyde (Wako) solution. We used Slow Fade Diamond Antifade Mountant for nuclear staining with DAPI (Invitrogen). The immunostained samples were visualized using fluorescence microscopy. The Hybrid Cell Count Software was utilized to analyze positive cells’ fluorescence intensity and percentage.

### Immunocytochemistry

In 24-well plates, 3 × 10^4^ cells were planted, and the cells were then treated with M-CSF and RANKL for 2 days. In the following incubation, the cells were fixed for 10 min at room temperature using a 4% paraformaldehyde (PFA) solution (Wako). Subsequently, 0.2% bovine serum albumin (BSA) and 0.3% Triton X-100 (Sigma-Aldrich) were used to block the cells for an h at room temperature. Antibodies against NRF2 (#Sc-365949, Santa Cruz Biotechnology) were treated with the cells for 3 h at a dilution 1:200 in 0.2% BSA. The samples were then exposed for an h at room temperature to Alexa Fluor 546 goat anti-mouse IgG2a cross-adsorbed secondary antibody (#A21133, Invitrogen). Slow Fade Diamond Antifade Mountant with DAPI (Invitrogen) was used to stain nuclei. Immunostaining was visualized using fluorescence microscopy. Blinded, five randomly chosen areas per glass coverslip were imaged. The fluorescence intensity and the percentage of positive cells on cells were quantified using Hybrid Cell Count Software, and the mean values were calculated.

### Statistics

All experiments in this study were repeated at least three times independently. All graphs show the data as the mean ± standard deviation. The Shapiro-Wilk test was conducted to determine whether the data distribution was normal. A two-tailed Student’s t-test was used to investigate two-group comparisons when the distribution was normal. A one-way or two-way analysis of variance was carried out for comparisons involving more than two groups, followed by Tukey’s post hoc test. The Kruskal–Wallis test was used for comparisons involving more than two groups when the distribution was non-normal, and Dunn’s post hoc test was used for two-group comparisons in those situations. Prism 9 software from GraphPad Software, La Jolla, CA, was used for all statistical calculations. *p* values < 0.05 was considered statistically significant.

### Supplementary information

Original western blots

## Data Availability

All data generated or analyzed in the course of this study are incorporated into this published article.
